# TEEG Induced A549 Cell Autophagy by Regulating the PI3K/AKT/mTOR Signaling Pathway

**DOI:** 10.1155/2019/7697610

**Published:** 2019-04-30

**Authors:** Lu Shi, Yijun Tu, Yu Xia, Siqi Ye, Chaozhi Ma, Yanwen Liu, Pengtao You

**Affiliations:** ^1^Department of Pharmacy, Medical College, Jianghan University, Hubei, Wuhan 430056, China; ^2^Hubei Key Laboratory of Resources and Chemistry of Chinese Medicine, Hubei University of Chinese Medicine, Hubei, Wuhan 430065, China; ^3^Department of Dermatology, The Second Affiliated Hospital of Guangzhou University of Chinese Medicine, Guangzhou, China

## Abstract

TEEG (3*β*,16*β*,23-trihydroxy-13,28-epoxyurs-11-ene-3-O-*β*-D-glucopyranoside) is derived from the chloroform extract of the Chinese medicine formula Shenqi San (CE-SS). In the present study, we aimed to elucidate the anticancer effect and possible molecular mechanism underlying the action of TEEG against the human non-small cell lung cancer (NSCLC) cell line A549 in vitro. A549 cells were incubated with different concentrations of TEEG. Cell proliferation was assessed by MTT assay. Autophagy was evaluated by immunofluorescence staining. Autophagy-associated proteins were examined by Western blot analysis. TEEG markedly inhibited A549 cell proliferation in a concentration-dependent manner. Immunofluorescence staining showed that TEEG induced autophagy in A549 cells. The LC3-II : LC3-I conversion ratio and the expression of Beclin-1, Atg5, Atg7, and Atg12 increased with the concentration of TEEG. In addition, increased TEEG concentration enhanced the expression of Class III p-PI3K and reduced the expression of Class I p-PI3K, p-AKT, p-mTOR, and p-P70S6K. These results indicate that TEEG induces autophagy of A549 cells through regulation of the PI3K/AKT/mTOR signaling pathway.

## 1. Introduction

Non-small cell lung cancer (NSCLC) is one of the most common malignant tumors of the respiratory system and is currently recognized as one of the leading causes of cancer-related deaths worldwide [1]. Although the advent of targeted therapies has improved outcomes in a subset of NSCLC patients, the overall 5-year survival rate remains less than 15%. Therefore, the identification of a safe and effective drug treatment for NSCLC is crucial [2, 3]. The significant improvement in the survival time of patients with lung carcinoma is ascribed to current therapeutic methodologies, including surgery, radiotherapy, and chemotherapy; however, the efficacy of overall treatment remains unsatisfactory [4]. Therefore, investigation of a reliable and clinically relevant alternative is a current focus of research.

Traditional Chinese medicine (TCM) has become increasingly popular in the West. The United States National Cancer Institute (NCI) spends around $120 million each year on research projects related to complementary and alternative medicine, including TCM [5, 6]. In recent years, studies of natural monomer products extracted from herbs have shown effectiveness in treating tumors, and a growing number of Chinese medicine formulas and extracts have been reported to have a reliable therapeutic effect on NSCLC [7–9].

Autophagy plays major roles in determining cellular fate, participating in development, cellular homeostasis, and both physiological and pathological processes. Autophagy is a discrete cellular process that is mediated by distinct groups of regulatory and executioner molecules [10, 11]. Previous studies revealed that inhibition of PI3K/AKT/mTOR signaling induces apoptosis and autophagy in A549 cells [12]. We have previously shown that the Chinese medicine formula Shenqi San (CE-SS) promotes A549 cell apoptosis and exerts analgesic effects in mice [13, 14]. We isolated TEEG (3*β*,16*β*,23-trihydroxy-13,28-epoxyurs-11-ene-3-O-*β*-D-glucopyranoside) by chloroform extraction from CE-SS and demonstrated that TEEG exerts a strong inhibitory effect on A549 cells. Although free radical scavenging and antimicrobial activities have been demonstrated previously [15], the biological activity of TEEG remains to be clarified. Importantly, the inhibitory effect of TEEG on A549 cells has not yet been reported. Therefore, in this study, we investigated the possible molecular mechanisms by which TEEG inhibits A549 cell proliferation.

## 2. Materials and Methods

### 2.1. Cell Culture

A549 cells were obtained from Wuhan University (Wuhan, China). The cells were cultured in RPMI-1640 medium (Gibco, MD, USA) supplemented with 10% fetal bovine serum (FBS) (Gibco, MD, USA) and 100 U of penicillin G with 100 *μ*g of streptomycin per ml. Cells were incubated at 37°C under 5% CO_2_ in a humidified atmosphere.

### 2.2. Cell Proliferation Assay

A549 cells were incubated in 96-well plates for 24 h at a density of 1.0 × 10^4^ cells per well with three secondary holes. After the cells were treated with different concentrations (1, 2, 4, 6, and 8 *μ*g/ml) of TEEG for 24 h, 10 *μ*l MTT (Sigma, MO, USA) was added to each well. After the cells were incubated for another 4 h, the supernatant was removed, and the formazan product obtained was dissolved in 150 *μ*l dimethylsulfoxide (DMSO) (Sigma) with stirring for 10 min on a QB-9001 Microporous Quick Shaker (Kylin-Bell Lab Instruments Co. Ltd., Jiangsu, China). The absorbance was read at 490 nm using a Spark 10M microplate reader (Tecan, Männedorf, Switzerland).

### 2.3. Immunofluorescence Staining

A549 cells were seeded into six-well plates (5 × 10^5^ cells/ml) and treated with 6 *μ*g/ml TEEG for 12 h. Cells were then fixed in 4% paraformaldehyde for 20 min followed by membrane permeabilization using 0.1% Triton X-100 for 30 min. After washing with PBS, slides were blocked with 5% BSA for 1 h at room temperature and subsequently incubated overnight at 4°C with Alexa Fluor® 488 Conjugated LC3I/II XP® Rabbit mAb (1 : 400) (Cell Signaling Technology, MA, USA). Cells were then stained with DAPI (1 *μ*g/ml) (Biosharp, China) for 5 min and washed three times with PBS. Finally, the signal was visualized using a confocal laser scanning microscope system (Leica TCS SP2, Germany).

### 2.4. Western Blot Analysis

The cells were seeded in six-well plates (2.0 × 10^5^ cells per well). After attachment, the cells were treated with different concentrations (1, 2, 4, and 6 *μ*g/ml) of TEEG for 6 h. The cells were then lysed, and protease inhibitor cocktail was added to obtain the whole protein content for quantification. The lysates were boiled for 5 min in protein loading buffer. The supernatants were collected, and proteins (40 *μ*g) were resolved by sodium dodecyl sulfate-polyacrylamide gel electrophoresis (SDS-PAGE; 12% gel). The separated proteins were then transferred to polyvinylidene fluoride membranes (Millipore, USA) and blocked in 5% nonfat milk for 2 h at room temperature. Membranes were then incubated overnight at 4°C with the following primary detection antibodies diluted in blocking buffer according to the manufacturer's instructions: anti-Class I p-PI3K (p85), anti-Class III p-PI3K (p85) (Abgent, Suzhou, China), anti-p-AKT (Ser473), anti-p-mTOR (Ser2448), anti-p-p70S6K (Thr389), anti-Class I PI3K (p85), anti-AKT, anti-P70S6K, anti-LC3-I/II, anti-Beclin-1, anti-Atg5, anti-Atg7, anti-Atg12, and anti-*β*-actin (Cell Signaling Technology). After washing three times with Tris-buffered saline (TBS) containing 0.5% (*v*/*v*) Tween-20, the membranes were incubated for 2 h at room temperature with HRP-conjugated secondary detection antibodies diluted in blocking buffer according to the manufacturer's instructions. After washing three times with TBS, blots were developed using an ECL detection reagent (Bio-Rad, USA) and analyzed using the FluorChem FC3 system (ProteinSimple, CA, USA).

### 2.5. Statistical Analysis

Statistical analysis was performed using SPSS Statistics 24.0 (IBM, NY, USA). Data were expressed as the mean ± standard deviation (SD) of values obtained from at least three independent experiments. The data were analyzed using one-way analysis of variance (ANOVA). A value of *P* < 0.05 was considered to denote a statistically significant difference.

## 3. Results

### 3.1. TEEG Decreases Proliferation of A549 Cells

**Figure 1:**
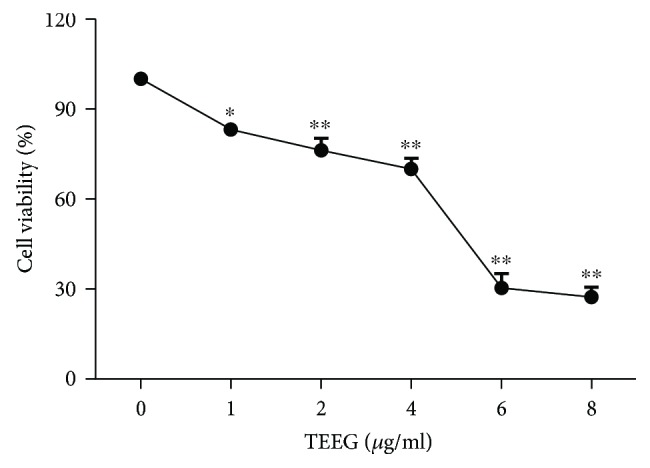
Effect of TEGG on the viability of A549 cells. After treatment with TEEG for 24 h, the viability of A549 cells was measured by MTT assay. ^∗^*P* < 0.05 and ^∗∗^*P* < 0.01 vs. control.

As shown in Figure 1, as the concentrations of TEEG increased, cell viability was significantly decreased in A549 cells compared with that in the control (*P* < 0.05 and *P* < 0.01). The half-maximal inhibitory concentration (IC_50_) of TEEG for A549 cells was 4.9 *μ*g/ml. Cell viabilities of A549 treated for 24 h with 1, 2, 4, 6, and 8 *μ*g/ml TEEG were 83.13%, 76.23%, 70.04%, 30.47%, and 27.35%, respectively. Thus, A549 cell viability decreased in a dose-dependent manner.

### 3.2. TEEG Induces A549 Cell Autophagy

**Figure 2:**
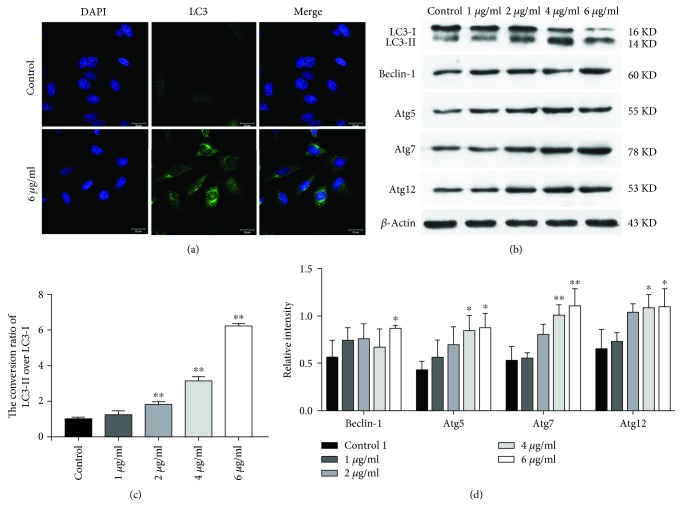
Effects of TEEG on the A549 cell autophagy. (a) LC3 expression and autophagosome formation were analyzed by confocal microscopy (200x). (b, c) Western blot analysis of the LC3-II : LC3-I conversion ratio in A549 cells. (b, d) Western blot analysis of autophagy-related protein expression in A549 cells. ^∗^*P* < 0.05 and ^∗∗^*P* < 0.01 vs. control.

Autophagosomes are spherical structures with double-layered membranes that participate in multistage processes involving many proteins. The conjugation leads to the conversion of the soluble form of LC3 (LC3-I) to the autophagic vesicle-associated form (LC3-II), which is used as a marker of autophagy [16, 17]. Through immunofluorescence detection, autophagosome formation can be visualized as LC3 accumulation in these organelles. Increased LC3-II expression was induced by TEEG (Figures 2(a) and 2(b)), which suggested that TEEG induces autophagy of A549 cells. To further confirm the induction of autophagy in TEEG-treated A549 cells, we investigated the LC3-II : LC3-I conversion ratio. In the treated groups (1, 2, 4, and 6 *μ*g/ml), the LC3-II : LC3-I conversion ratios were 1.2-, 1.8-, 3.1-, and 6.2-fold higher, respectively, than that in the control group (Figure 2(c)). As shown in Figures 2(b) and 2(d), Beclin-1 was significantly upregulated in the treated group (6 *μ*g/ml; *P* < 0.05), while Atg5, Atg7, and Atg12 were significantly downregulated (4 and 6 *μ*g/ml; *P* < 0.01 and *P* < 0.05). These results indicated that TEEG inhibits the proliferation of A549 cells by inducing autophagy.

### 3.3. PI3K/AKT/mTOR Pathway Is Involved in TEEG-Induced Autophagy

**Figure 3:**
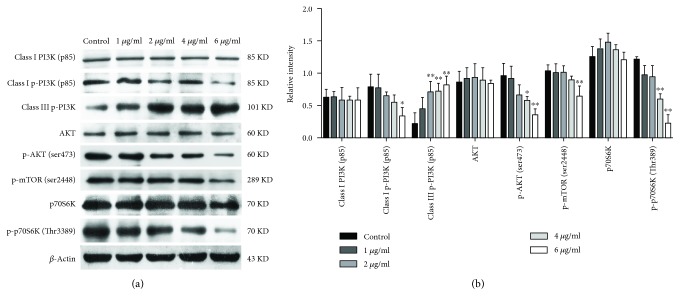
The PI3K/Akt/mTOR pathway is involved in TEEG-induced autophagy. (a, b) Western blot analysis of the levels of Class I PI3K, Class I p-PI3K, Class III p-PI3K, AKT, p-AKT, p-mTOR, p70S6K, and p-p70S6K in A549 cells was treated with TEEG for 6 h. ^∗^*P* < 0.05 and ^∗∗^*P* < 0.01 vs. control.

The PI3K/AKT/mTOR signaling pathway had been demonstrated to be involved in autophagy [12]. To investigate the involvement of the PI3K/AKT/mTOR pathway in TEEG-induced autophagy, we analyzed the expression of autophagy-related proteins in A549 cells treated with TEEG by Western blotting. As shown in Figure 3, the expression of Class III p-PI3K was significantly upregulated in TEEG-treated groups compared to that in the control group (*P* < 0.01). In contrast, the levels of Class I p-PI3K and p-mTOR were significantly downregulated in cells treated with 6 *μ*g/ml TEEG (*P* < 0.05 and *P* < 0.01), and the levels of p-AKT and p-P70S6K were significantly downregulated in the cells treated with 4 and 6 *μ*g/ml TEEG (*P* < 0.05 and *P* < 0.01, respectively). However, the levels of Class I PI3K, AKT, and p70S6K were unchanged by TEEG treatment. These results indicated that the PI3K/AKT/mTOR pathway is involved in TEEG-induced autophagy in A549 cells.

## 4. Discussion

Natural products have long been used widely as a significant source of therapeutically effective drugs, and their importance in the prevention and treatment of tumors is becoming increasingly evident [18]. In addition, an increasing number of Chinese herbal medicines and extracts have been shown to exhibit anti-inflammatory, antioxidative, and antiliver fibrosis and anticancer effects [19–22]. These findings suggest that Chinese herbal medicines and extracts have great potential in the treatment of many diseases.

Autophagy is a type II cell death a process involved in the isolation of cellular organelles, long-lived proteins, and cytoplasmic parts and leading to the formation of autophagosomes. This double-membraned structure fuses with a lysosome to form a modified structure known as the autolysosome, which is ultimately degraded [23, 24]. In this study, immunofluorescence detection of autophagy-related factors revealed that TEEG enhances LC3 expression, suggesting that TEEG inhibits A549 cell proliferation by inducing autophagy. The formation of autophagosomes occurs via two pathways: the Atg12-Atg5-Atg16 pathway and the Atg4-Atg7-Atg3 pathway. Conjugations lead to the conversion of the soluble form of LC3 (LC3-I) to the autophagic vesicle-associated form (LC3-II), which is used as a marker of autophagy [25]. The LC3-II : LC3-I conversion ratio is used to evaluate the level of autophagy of NSCLC [26, 27]. Moreover, our subsequent investigations demonstrated the ability of TEEG to upregulate levels of Beclin-1, Atg5, Atg7, and Atg12 and increase the LC3-II : LC3-I conversion ratio. These findings suggested that TEEG induces autophagy in A549 cells via both the Atg12-Atg5-Atg16 and Atg4-Atg7-Atg3 pathways to increase the formation of autophagosomes and regulate the expression of autophagy-related proteins; however, the specific mechanism requires further investigation.

The PI3K/AKT/mTOR pathway is essential for the regulation of growth, proliferation, cell cycle, metastasis, apoptosis, and autophagy [28–30]. Autophagy is also regulated by PI3K type III, which is a component of a multiprotein complex that includes Beclin-1. The PI3Ks (Class I and Class III) are a family of enzymes that are involved in autophagy signaling. Class III PI3Ks have been shown to stimulate autophagy. Generally, activation of the Class I PI3Ks suppresses autophagy via the well-established PI3K/AKT/mTOR (mechanistic target of rapamycin) complex 1 (mTORC1) pathway [31–33]. In this study, we showed that TEEG treatment suppressed the phosphorylation of Class I PI3K and promoted the phosphorylation of Class III PI3K in A549 cells. This suggested that TEEG induces autophagy in A549 cells by regulating both Class I PI3K and Class III PI3K. The regulation of autophagy is a highly complex process involving many signaling complexes and pathways, with TOR (mTOR in mammals) as the central player [34]. As a member of the PI3K-related kinase family, mTOR is associated with cell proliferation and cell metabolism. Previous studies have shown that inhibition of AKT and its downstream target mTOR contributes to the initiation of autophagy [35]. Meanwhile, mTOR is an important downstream target of PI3K/AKT and positively regulates p70S6K; PI3K/AKT/mTOR is an important intracellular signaling pathway in regulating cell survival and death. Signaling through the PI3K/AKT/mTOR pathway controls proliferation, apoptosis, and autophagy of cells [36, 37]. This is consistent with our results showing that TEEG suppressed the phosphorylation of AKT, mTOR, and p70S6K expression in A549 cells. Our results suggested that TEEG induced autophagy in A549 cells by regulating the PI3K/AKT/mTOR signaling pathway.

In conclusion, we conclude that TEEG inhibits A549 proliferation by inducing autophagy via the PI3K/AKT/mTOR signaling pathway. Therefore, the results of the current study further clarify the mechanism underlying the antitumor effects of TEEG; however, further studies are required for a comprehensive elucidation of the mechanism.

## Data Availability

The data used to support the findings of this study are available from the corresponding author upon request.

## References

[1] You J., Cheng J., Yu B., Duan C., Peng J. (2018). Baicalin, a Chinese herbal medicine, inhibits the proliferation and migration of human non-small cell lung carcinoma (NSCLC) cells, A549 and H1299, by activating the SIRT1/AMPK signaling pathway. *Medical Science Monitor*.

[2] Tsai M. F., Wang C. C., Chen J. J. (2014). Tumour suppressor HLJ1: a potential diagnostic, preventive and therapeutic target in non-small cell lung cancer. *World Journal of Clinical Oncology*.

[3] Han Y., Song X., Shi K., Zhou S. J., Yu D. P., Liu Z. D. (2016). Clinicopathological significance and a potential drug target of RAR&beta; in non-small-cell lung carcinoma: a meta-analysis and a systematic review. *Drug Design, Development and Therapy*.

[4] Zhu B., Yang J. R., Jia Y. (2017). Overexpression of NR4A1 is associated with tumor recurrence and poor survival in non-small-cell lung carcinoma. *Oncotarget*.

[5] Xu W., Yang G., Xu Y. (2014). The possibility of traditional chinese medicine as maintenance therapy for advanced nonsmall cell lung cancer. *Evidence-Based Complementary and Alternative Medicine*.

[6] Man S., Gao W., Zhang Y. (2009). Antitumor and antimetastatic activities of Rhizoma Paridis saponins. *Steroids*.

[7] Zhao R., Chen M., Jiang Z. (2015). Platycodin-D induced autophagy in non-small cell lung cancer cells via PI3K/Akt/mTOR and MAPK signaling pathways. *Journal of Cancer*.

[8] Xu L., Meng X., Xu N. (2018). Gambogenic acid inhibits fibroblast growth factor receptor signaling pathway in erlotinib-resistant non-small-cell lung cancer and suppresses patient-derived xenograft growth. *Cell Death & Disease*.

[9] Chen M., Hu C., Guo Y. (2018). Ophiopogonin B suppresses the metastasis and angiogenesis of A549 cells in vitro and in vivo by inhibiting the EphA2/Akt signaling pathway. *Oncology Reports*.

[10] Maiuri M. C., Zalckvar E., Kimchi A., Kroemer G. (2007). Self-eating and self-killing: crosstalk between autophagy and apoptosis. *Nature Reviews Molecular Cell Biology*.

[11] Eisenberg-Lerner A., Bialik S., Simon H. U., Kimchi A. (2009). Life and death partners: apoptosis, autophagy and the cross-talk between them. *Cell Death & Differentiation*.

[12] Zhang Q., Zhu H., Xu X., Li L., Tan H., Cai X. (2015). Inactivated Sendai virus induces apoptosis and autophagy via the PI3K/Akt/mTOR/p70S6K pathway in human non-small cell lung cancer cells. *Biochemical and Biophysical Research Communications*.

[13] Xia Y., Shi L., Ai Z. Z., Zhang D. Z., Liu Y. W., You P. T. (2017). Chinese medicine formula “Shenqi San” extract inhibits proliferation of human lung adenocarcinoma A549 cells via inducing apoptosis. *Current Medical Science*.

[14] Shi L., Ai Z. Z., Zhang D. Z., Qin J., Chen C., Liu Y. (2016). Anti-tumor and analgesic effects of Shenqi powder. *Medical Journal of Wuhan University*.

[15] Mencherini T., Picerno P., Scesa C., Aquino R. (2007). Triterpene, Antioxidant, and Antimicrobial Compounds from *Melissa officinalis*. *Journal of Natural Products*.

[16] Ohsumi Y. (2001). Molecular dissection of autophagy: two ubiquitin-like systems. *Nature Reviews Molecular Cell Biology*.

[17] Nishida K., Kyoi S., Yamaguchi O., Sadoshima J., Otsu K. (2009). The role of autophagy in the heart. *Cell Death & Differentiation*.

[18] Deng R., Tang J., Xia L. P. (2009). ExcisaninA, a diterpenoid compound purified from Isodon MacrocalyxinD, induces tumor cells apoptosis and suppresses tumor growth through inhibition of PKB/AKT kinase activity and blockade of its signal pathway. *Molecular Cancer Therapeutics*.

[19] Xia Y., Yu B., Ma C. (2018). Yu Gan Long reduces rat liver fibrosis by blocking TGF-*β*1/Smad pathway and modulating the immunity. *Biomedicine & Pharmacotherapy*.

[20] You P., Fu S., Yu K. (2018). Scutellarin suppresses neuroinflammation via the inhibition of the AKT/NF-*κ*B and p38/JNK pathway in LPS-induced BV-2 microglial cells. *Naunyn-Schmiedeberg's Archives of Pharmacology*.

[21] Cui Y., Li C., Zeng C. (2018). Tongmai Yangxin pills anti-oxidative stress alleviates cisplatin-induced cardiotoxicity: network pharmacology analysis and experimental evidence. *Biomedicine & Pharmacotherapy*.

[22] Su X., Li Y., Jiang M. (2019). Systems pharmacology uncover the mechanism of anti-non-small cell lung cancer for Hedyotis diffusa Willd. *Biomedicine & Pharmacotherapy*.

[23] Mizushima N., Klionsky D. J. (2007). Protein turnover via autophagy: implications for metabolism. *Annual Review of Nutrition*.

[24] Gugnoni M., Sancisi V., Manzotti G., Gandolfi G., Ciarrocchi A. (2016). Autophagy and epithelial-mesenchymal transition: an intricate interplay in cancer. *Cell Death & Disease*.

[25] Xiao J., Zhu X., Kang B. (2015). Hydrogen sulfide attenuates myocardial hypoxia-reoxygenation injury by inhibiting autophagy via mTOR activation. *Cellular Physiology and Biochemistry*.

[26] Ma J., Wu K., Liu K., Miao R. (2018). Effects of MALAT1 on proliferation and apo- ptosis of human non-small cell lung cancer A549 cells in vitro and tumor xenograft growth in vivo by modulating autophagy. *Cancer Biomarkers*.

[27] Liu F., Gao S., Yang Y. (2018). Antitumor activity of curcumin by modulation of apoptosis and autophagy in human lung cancer A549 cells through inhibiting PI3K/Akt/mTOR pathway. *Oncology Reports*.

[28] Chen J. (2012). Roles of the PI3K/Akt pathway in Epstein-Barr virus-induced cancers and therapeutic implications. *World Journal of Virology*.

[29] Chen Y. L., Law P. Y., Loh H. H. (2005). Inhibition of PI3K/Akt signaling: an emerging paradigm for targeted cancer therapy. *Current Medicinal Chemistry-Anti-Cancer Agents*.

[30] Hennessy B. T., Smith D. L., Ram P. T., Lu Y., Mills G. B. (2005). Exploiting the PI3K/AKT pathway for cancer drug discovery. *Nature Reviews Drug Discovery*.

[31] Axe E. L., Walker S. A., Manifava M. (2008). Autophagosome formation from membrane compartments enriched in phosphatidylinositol 3-phosphate and dynamically connected to the endoplasmic reticulum. *The Journal of Cell Biology*.

[32] Liu M., Ma S., Liu M. (2014). Synergistic killing of lung cancer cells by cisplatin and radiation via autophagy and apoptosis. *Oncology Letters*.

[33] Yu X., Long Y. C., Shen H. M. (2015). Differential regulatory functions of three classes of phosphatidylinositol and phosphoinositide 3-kinases in autophagy. *Autophagy*.

[34] Ferraro E., Cecconi F. (2007). Autophagic and apoptotic response to stress signals in mammalian cells. *Archives of Biochemistry and Biophysics*.

[35] Huo R., Wang L., Liu P. (2016). Cabazitaxel-induced autophagy via the PI3K/Akt/mTOR pathway contributes to A549 cell death. *Molecular Medicine Reports*.

[36] Zhang L., Wang H., Xu J., Zhu J., Ding K. (2014). Inhibition of cathepsin S induces autophagy and apoptosis in human glioblastoma cell lines through ROS-mediated PI3K/AKT/mTOR/p70S6K and JNK signaling pathways. *Toxicology Letters*.

[37] Guertin D. A., Sabatini D. M. (2007). Defining the role of mTOR in cancer. *Cancer Cell*.

